# The effects of gender discrimination on medical students‘ choice of specialty for their (junior) residency – a survey among medical students in Germany

**DOI:** 10.1186/s12909-024-05579-9

**Published:** 2024-05-30

**Authors:** Jule Stock, Andrea Kaifie

**Affiliations:** 1 Institute for Occupational, Social, and Environmental Medicine, Medical Faculty, RWTH Aachen University, Pauwelsstrasse 30, 52074 Aachen, Germany; 2 Institute and Outpatient Unit for Occupational, Social, and Environmental Medicine, FAU Erlangen-Nuremberg, Henkestrasse 9-11, 91054 Erlangen, Germany

**Keywords:** Medical education, Sexism, Harassment, Degradation

## Abstract

**Background:**

Gender discrimination is known to affect societies in many different settings. Medical education is no exception. This study focusses on the consequences, gender discrimination can have on medical students and their choice of (junior) residency specialty.

**Methods:**

An online questionnaire was developed and distributed among the 40 medical faculties in Germany. The study population contained medical students in their fifth and sixth academic year.

**Results:**

The survey’s participants consisted of 759 students from 31 universities. Female medical students experienced significantly more gender discrimination compared to their male colleagues (f = 487, 87.9% vs. m = 76, 45.8%, *p* < 0.0001). The specialties with the most reported gender discrimination were family medicine (f = 180, 42.9% vs. m = 15, 23.8%, *p* < 0.05), followed by surgery (f = 369, 87.4% vs. m = 44, 69.8%, *p* < 0.05), internal medicine (f = 282, 67.3% vs. m = 37, 58.7%, ns), orthopaedics/casualty surgery (f = 270, 65.1% vs. m = 32, 50.8%, *p* < 0.05), and gynaecology (women (f = 142, 34.1% vs. m = 34, 54.0%, *p* < 0.05). Gynaecology was the only specialty, men experienced more discrimination compared to women. Among the students that ever changed their specialty of choice (f = 346 (73.3%) m = 95 (72%)), significantly more women than men claimed gender discrimination to be one of the main three reasons for their specialty choice (f = 42, 12.1% vs. m = 1, 1.1%, *p* < 0.05). In addition, 53 students (f = 50 (10.6%) m = 3 (2.3%)) stated to rule out a specialty from the beginning due to gender discrimination.

**Conclusion:**

Gender discrimination is frequently experienced by medical students in Germany. It influences their choice of medical specialty directly. Our data suggest a fundamental problem that proposes and implicates certain specialties to be attractive for only one gender.

**Supplementary Information:**

The online version contains supplementary material available at 10.1186/s12909-024-05579-9.

## Background

Since they have first been accepted to German medical universities in the last years of the 19th century, the number of female medical students has increased over the time. While in 1993 45.7% (41,417) of the medical students in Germany were women, they have obtained the majority of 64.36% (69,597) of the total 108,130 medical university places in 2022 [1]. The admission criteria of German medical schools might be of interest as one possible reason for this feminization of the medical field. Apart of some exceptions, there are three main centrally managed admission categories. Students can be admitted either based on the final school grade, the time of waiting for admission or the university’s choice (often based on grades, additional tests or interviews). Since the average of female students tend to reach slightly higher final grades than their male fellow students (f = 2.27 vs. m = 2.45) [2], they might nowadays secure a place in medical school more easily. However, that does not explain the worldwide increase of women in the medical field [3, 4], as the admission criteria to medical schools differ between the countries. For Germany, a representative from an official medical association came to the conclusion that the high workload and responsibility and the worse pay in comparison to less responsible jobs might be a reason for the feminization as it became less attractive for men [5].

This high percentage of women in medical schools is however not represented in every medical specialty. Statistics of resident German doctors show, that women are far less often found in the fields of urology, orthopaedics and surgery, while less men practice as paediatricians or psychiatrists [6].

That raises the question, which factors influence the medical students’ decision, when it comes to choosing their medical specialty. There are significant differences on specialty choices between female and male students, especially for paediatrics, orthopaedics and surgery [7]. The lack of role models of the own gender in the different specialties might be one reason [8], making the genders’ predominance in certain specialties a self-fulfilling prophecy. A study, that has been carried out in the US, identifies gender discrimination as another influencing factor on American medical students’ specialty and residency choice [9].

Gender discrimination is a frequently experienced phenomenon amongst medical students [10–15], that is known to have negative psychological impacts, such as an increased risk of depression, anxiety, emotional exhaustion or substance abuse [11]. A German pilot study in the field of gender discrimination at a medical school indicated, that gender discrimination can lead to negative perceptions of ones’ opportunities, as well [10]. Their research led the authors of this study believe, that gender discrimination, that was predominantly experienced by women, can have influence on the students’ future career path. However, the nature of this influence was not specified.

To contribute against inequality in medical education, this study is going to examine the gender discrimination experiences of medical students throughout Germany and evaluate whether it has a significant influence on the specialty choice and therefore the predominance of one gender in certain specialties.

## Methods

### Development of questionnaire

The online questionnaire developed contained 34 questions and was mainly adapted from pre-existing questionnaires that have already been used in the field [10, 16]. The participants were divided into two answer categories, one asking about experienced gender discrimination and one about witnessed gender discrimination. Prior to the questions about gender discrimination, the questionnaire gave a short definition of gender discrimination and microaggressions to sensitize the participants and to help them categorise their experienced or witnessed incidents of discrimination. For some variables, examples were given for explanatory reasons. For example: “Unwanted persistent affections (e.g. flirting, asking on dates)”, “public humiliation (e.g. to show up somebody on rounds)”.

The questionnaire was divided into sections including:

- Sociodemographic data: university, phase of studies, gender, age (religion and parents’ highest educational qualification were optional).
- Current specialty choice and desirable work qualities: qualities had to be assessed with a 4-point Likert scale “unimportant, less important, important, very important”.
- Experienced or witnessed gender discrimination: form, aggressors, setting.
- Perceptions of dis-/advantage because of gender.
- Changes made concerning the specialty of choice and their reasons.
- Receiving advise for/against choosing a specialty due to the gender.

When asking about forms, setting or aggressors of discrimination, a five-point Likert scale was used, containing the values “never, rarely, occasionally, often, very often”. The questions concerning gender discrimination were adapted from the questionnaire used in the research article of Jendretzky et al. [10]. The items, inquiring the specialty choices and desirable work qualities, were partially used from the “Berufsmonitoring Medizinstudierender 2018” [16], which questioned approximately 13000 medical students in Germany on work expectations and career goals. For settings or forms of gender discrimination, it was possible to give multiple answers due to possible multiple experienced or witnessed incidents. We pretested the questionnaire in a group of 15 voluntary participants and added small alterations in the final quationnaire.

### Data collection

Data was collected over a period of 44 days from 2023/01/16 to 2023/02/28. For the questionnaire distribution and data assembly we used SoSci Survey [17], accessible for the participants via the website www.soscisurvey.de. The link for the questionnaire was distributed among 40 German medical faculties by E-Mail, WhatsApp, Instagram and online fora. To increase the response rate, the faculties were asked to post reminders to the survey after about 25 days, what led to another small peak of responses. Altogether, students from 31 medical faculties filled out the questionnaire. To rule out participation bias, the study’s focus on gender discrimination was not apparent in the cover letter. It was introduced after the sociodemographic data and specialty choice questions. The cover letter stated, that the students will be participating in “a survey to figure out, which factors influence medical students’ decision when it comes to choosing a specialty for the junior-/residency.”

### Statistical analysis

The data collected via soscisurvey.de was downloaded and processed in Excel. The questionnaire’s Likert scale values of the questions concerning:

- the experienced or witnessed discrimination (whether it ever happened, forms, setting and specialties).
- regarding the own gender as dis-/advantage.
- the gender of the aggressors.
- feeling like having to put in more effort compared to the other gender.

were dichotomised into “never” or “ever” experienced. The queried desired work qualities were dichotomised into “less important” (un- and less important) or “important” ((very) important). Due to the small total number of participants that identified as diverse (*n* = 8), the group was not included in descriptive statistics or tests for statistical significance. The statistical analysis was carried out with the use of “SAS OnDemand for Academics”. The categorical variables were analysed using frequencies. The Chi^2^-Test was used to determine significant differences between the male and female students, indicating statistical significance with a value of *p* < 0.05.

## Results

### Participants

The participants (Table 1) of this study included medical students from 31 of 40 German medical faculties from the ninth semester to the practical year (the last academic year in German medical school) covering enrolled students from the 5th and 6th year. 759 participants were matching these criteria, 603 of them completed the questionnaire. The ratio between the gender categories in the study population differed from that of all German medical students with a higher proportion of female students in our study (f = 63.8%, m = 36.2%, d = no data for Germany [1] vs. f = 76.0%, m = 22.9%, diverse = 1.1% in our study population).

**Table 1:** Participants’ sociodemograohic data and current specialties of choice

	female	male	diverse	Total
Participants	577 (76.0%)	174 (22.9%)	8 (1.1%)	759 (100%)
Mean age (years)	25.4	26.6	26.5	25.7
**Phase of Studies**				
9. Semester	230	65	2	297
10. Semester	37	6	3	46
Second state exam ahead	105	25	1	131
Second state exam passed	29	8	0	37
Junior residency	176	70	2	248
**Current specialty of Choice**	**f = 559**	**m = 165**		*n* = 724
Family medicine	57 (10.2%)	8 (4.9%)		65 (8.9%)
Anaesthesiology	79 (14.1%)	37 (22.4%)		116 (16.0%)
Ophthalmology	11 (2.0%)	3 (1.8%)		14 (1.9%)
Surgery	40 (7.2%)	9 (5.5%)		49 (6.8%)
Gynaecology	69 (12.3%)	2 (1.2%)		71 (9.8%)
Otorhinolaryngology	9 (1.6%)	2 (1.2%)		11 (1.5%)
Dermatology	13 (2.3%)	0 (0%)		13 (1.8%)
Internal medicine	53 (9.5%)	28 (17.0%)		81 (11.2%)
Paediatrics	60 (10.7%)	12 (7.3%)		72 (9.9%)
Neurology	40 (7.1%)	12 (7.3%)		52 (7.2%)
Psychiatry	42 (7.5%)	10 (6.1%)		52 (7.2%)
Radiology	17 (3.0%)	10 (6.1%)		27 (3.7%)
Orthopaedics	13 (2.3%)	12 (7.3%)		25 (3.5%)
Urology	8 (1.4%)	5 (3.0%)		13 (1.8%)
Other	48 (8.6%)	15 (9.1%)		63 (8.7%)

### Current specialty of choice and desirable work qualities

In addition to the students` current specialty of choice (Table 1), participants were asked to state the personal importance of certain statements regarding their future work. We observed significant differences between men and women in four of the twelve categories. Women found “flexible working hours” (f = 381, 68.8% vs. m = 92, 56.4%, *p* = 0.0035) as well as “regular working hours” (f = 462, 83.2% vs. m = 124, 75.6%, *p* = 0.027) and “knowing not only the medical history, but also the life story of a patient” (f = 353, 63.6% vs. m = 78, 47.6%, *p* = 0.0002) significantly more important compared to men. In.

the group of men, “good career options” (*n* = 110, 67.5%) were regarded as significantly more important compared to women (*n* = 273, 49,4%, *p* < 0.0001). Most often considered as important (86.4%) was “achieving a good work-life balance”, with no statistically significant difference between men and women.

### Gender discrimination

Gender discrimination among medical students during their academic career was experienced significantly more often by women than men (f = 487, 87.9% vs. m = 76, 45.8%, *p* < 0.0001). Men, that never experienced gender discrimination themselves, stated more often to have witnessed gender discrimination than the women that have never been discriminated (f = 25, 37.9% vs. m 64, 72.7%).

**Fig. 1:**
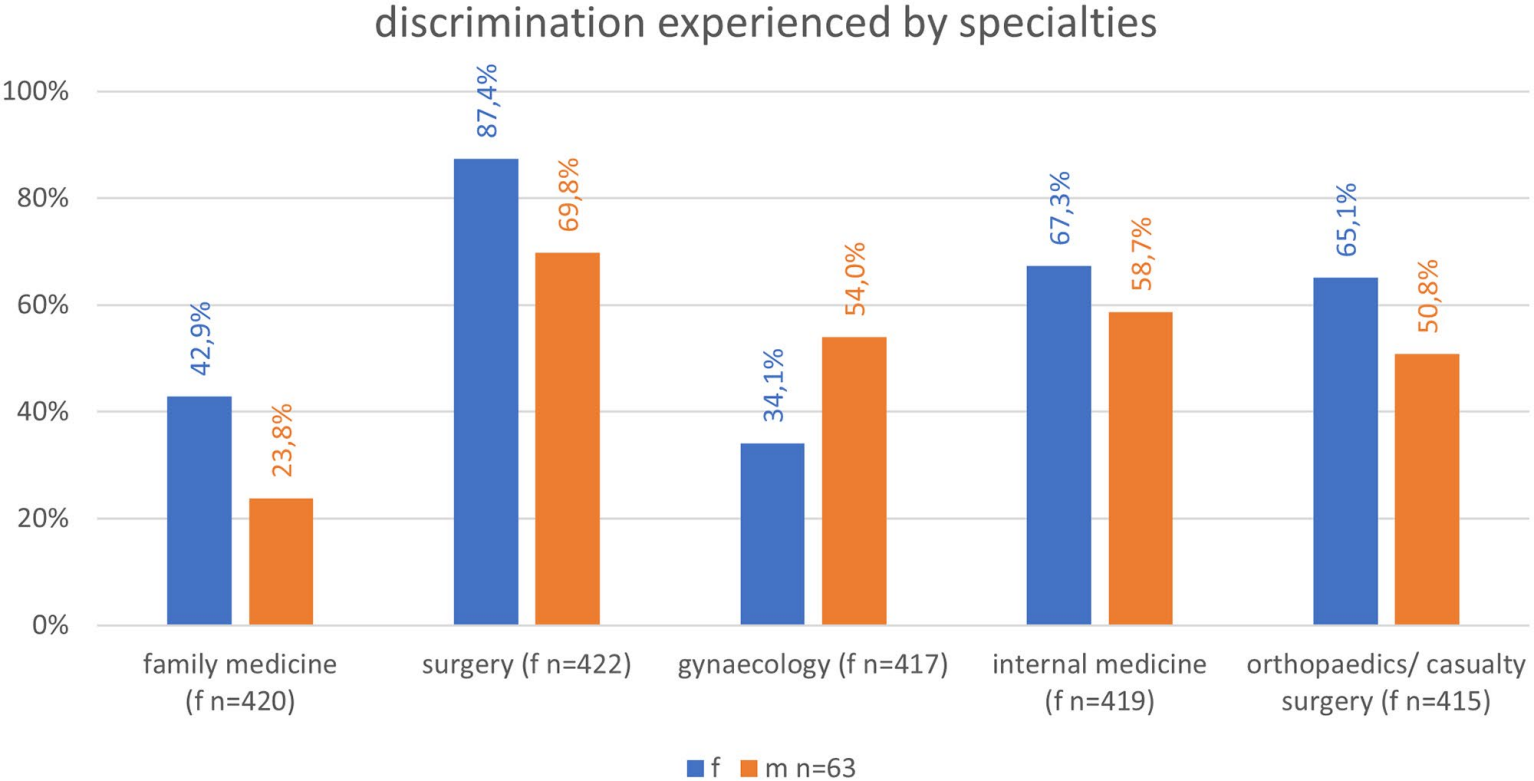
The five most common specialties to experience gender discrimination itemised by gender

As shown in Fig. 1, the specialties, women mostly experienced gender discrimination in were family medicine (f = 180, 42.9% vs. m = 15, 23.8%, *p* = 0.0041), surgery (f = 369, 87.4% vs. m = 44, 69.8%, *p* = 0.0002), internal medicine (f = 282, 67.3% vs. m = 37, 58.7%, ns) and orthopaedics/casualty surgery (f = 270, 65.1% vs. m = 32, 50.8%, *p* = 0.0287). Except for internal medicine, gender discrimination, experienced by men, was significantly less frequent compared to women. Men stated to have been discriminated in gynaecology significantly more often than women (f = 142, 34.1% vs. m = 34, 54.0%, *p* = 0.0022).

Table 2 shows the settings where *n* = 560 students were either discriminated themselves or witnessed gender discrimination. The three most often selected settings were nursing practice internship (*n* = 465, 83%), clinical traineeships (*n* = 464, 82.9%) and university lectures and classes (*n* = 434, 77.5%). Altogether, *n* = 47 (8.4%) of the participants reported to have experienced gender discrimination in the nursing practice internship “very often”. 78.1% (*n* = 139) of students in their junior residency witnessed or experienced gender discrimination in this setting.

Experienced as well as witnessed forms of gender discrimination can be found in Table 2. Women have experienced gender discrimination mainly in form of degrading jokes and insults (f = 409, 93.4% vs. m = 45, 69.2%, *p* < 0.0001), degrading gestures (f = 251, 57.3% vs. m = 25, 38.5%, *p* = 0.0044), degrading nicknames (f = 389, 88.8% vs. m = 49, 75.4%, *p* = 0.0021), unfounded questioning of one’s knowledge (f = 347, 79.2% vs. m = 38, 58.5%, *p* = 0.0002), unwanted touch (f = 266, 60.7% vs. m = 28, 43.1%, *p* = 0.007) and unwanted inappropriate compliments (f = 329, 75.1% vs. m = 33, 50.8%, *p* < 0.0001) significantly more often compared to the male students. Male students reported to have experienced, more insults and verbal abuse (f = 31.5% vs. m = 35.4%, n.s.) and public humiliation (f = 39.7% vs. m = 41.5, n.s.).

**Table 2:** Reported incidents of gender discrimination by male and female students. Specified by setting, form and aggressors’ gender

Setting of discrimination (experienced and witnessed)	*n* = 560 (multiple answers possible)			
University lectures and classes	434 (77.5%)			
Nursing practice internship	465 (83.0%)			
Clinical traineeship	464 (82.9%)			
Internships	383 (68.4%)			
Work at a hospital ward	257 (45.9%)			
Junior residency (*n* = 178)	139 (78.1%)			
**Forms of discrimination**	***Female = 438***	***Male = 65***	***Witnessed = 78***	***p-value***
degrading jokes/insults	409 (93.4%)	45 (69.2%)	61 (78.2%)	< 0.0001
degrading gestures concerning the gender	251 (57.3%)	25 (38.5%)	28 (35.9%)	= 0.0044
degrading nicknames	389 (89.0%)	49 (75.4%)	56 (71.8%)	= 0.0021
unfounded questioning of one’s knowledge	347 (79.2%)	38 (58.5%)	50 (64.1%)	= 0.0002
insults, verbal abuse	138 (31.5%)	23 (35.4%)	13 (16.7%)	n.s.
threats, harassment	161 (36.8%)	18 (27.7%)	14 (17.4%)	n.s.
unwanted touch	266 (60.7%)	28 (43.1%)	24 (30.8%)	= 0.007
favouring fellow students of the other gender	325 (74.2%)	48 (73.9%)	35 (44.9%)	n.s.
public humiliation	174 (39.7%)	27 (41.5%)	25 (32.1%)	n.s.
social exclusion	120 (27.5%)	16 (24.6%)	7 (9.0%)	n.s.
unwanted showing of pornographic material	18 (4.1%)	4 (6.2%)	1 (1.3%)	n.s.
unwanted persistent affections	142 (32.5%)	14 (21.5%)	15 (19.2%)	n.s.
unwanted inappropriate compliments	329 (75.3%)	33 (50.8%)	33 (42.3%)	< 0.0001
subliminal sexual extortion	17 (3.9%)	4 (6.2%)	1 (1.3%)	n.s.
sexual harassment / assault	26 (5.7%)	4 (6.2%)	0 (0%)	n.s.
**Aggressors**	***F = 419/420***	***M = 62***		
Same gender as discriminated	245 (58.3%) *n* = 419	51 (82.3%)		= 0.0003
Other gender than discriminated	413 (98.6%) *n* = 420	56 (90.3%)		= 0.0001
**Aggressors advising to/against a specialty**	***F = 469*** *Advising to*	***M = 130*** *Advising to*	***F = 469*** *Advising against*	***M = 130*** *Advising against*
Fellow students	49 (10.5%)	4 (3.1%)	82 (17.5%)	9 (6.9%)
Lecturers	82 (17.5%)	2 (1.5%)	84 (17.9%)	5 (3.9%)
Junior physicians	110 (23.5%)	12 (9.2%)	136 (29.0%)	7 (5.4%)
Senior physicians	142 (30.3%)	9 (6.9%)	150 (32.0%)	3 (2.3%)
Chief physicians	61 (13.0%)	3 (2.3%)	68 (14.5%)	2 (1.5%)
Family members	91 (19.4%)	3 (2.3%)	83 (17.7%)	7 (5.4%)
Medical technical assistants	17 (3.6%)	0 (0%)	16 (3.4%)	2 (1.5%)
Nurses	35 (7.5%)	5 (3.9%)	55 (11.7%)	6 (4.6%)
other	42 (8.9%)	2 (1.5%)	41 (8.7%)	8 (6.2%)
**Regarding the own Gender as**		***F n = 472***	***M n = 132***	***p-value***
	a disadvantage	387 (82%)	64 (48.5%)	< 0.0001
	an advantage	336 (71.2%)	90 (68.2%)	n.s.
**Changes in the specialty-choice**		***F n = 472***	***M n = 131***	
Not considered a speicatly in the first place	Due to gender discrimination	50 (10.6%)	3 (2.3%)	
	Other reasons	312 (66.1%)	91 (69.5%)	
redecided on the specialty choice	Ever	152 (32.2%)	44 (33.3%) *n* = 132	
	Multiple times	194 (41.1%)	51 (38.6%) *n* = 132	

**Fig. 2:**
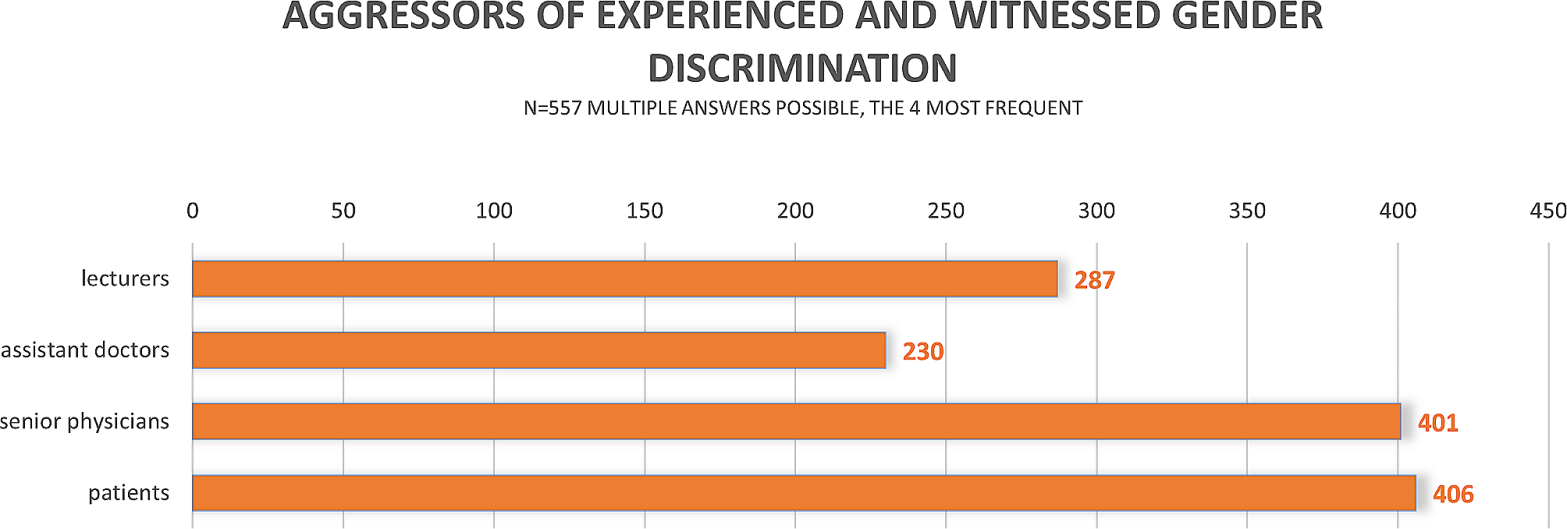
Most frequently named aggressors of combined, sum of experienced and witnessed gender discrimination incidents

The main aggressor of experienced and witnessed gender discrimination were patients (*n* = 406, 72.9%) followed by senior physicians (*n* = 401, 72%) *(*Fig. 2*).*

In addition, participants were asked how often they experienced gender discrimination by aggressors of their own gender and/or of the other gender (Table 2). Men were significantly more often discriminated by their own gender compared to women (women discriminated by women: *n* = 245, 58.3% vs. men discriminated by men: *n* = 51, 82.3%, *p* = 0.0003). In contrast, women were significantly more often discriminated by the other gender (women discriminated by men: *n* = 413, 98.6% vs. men discriminated by women: *n* = 56, 90.32%, *p* = 0.0001).

Furthermore, participants were asked whether they have ever perceived their gender as an advantage or disadvantage during their study of medicine (Table 2). There was no significant difference between the perception of their gender as an advantage between male and female students (f = 336, 71.2% vs. m = 90, 68.2%, n.s.). In contrast, the female students stated significantly more often, that they frequently assessed their gender as a disadvantage, than men (f = 387, 82% vs. m = 64, 48.5%, *p* < 0.0001).

### Changes in the desired specialty

The medical students were also asked, whether they have ever changed their specialty choice during their medical education. Among the female students, *n* = 346 (73.3%) reported to have changed their specialty choice, *n* = 194 (41.1%) of them even multiple times. Male students had similar percentages with *n* = 95 (72%) ever changing their specialty choice and *n* = 51 (38.6%) reconsidering multiple times. (Table 2).

**Fig. 3:**
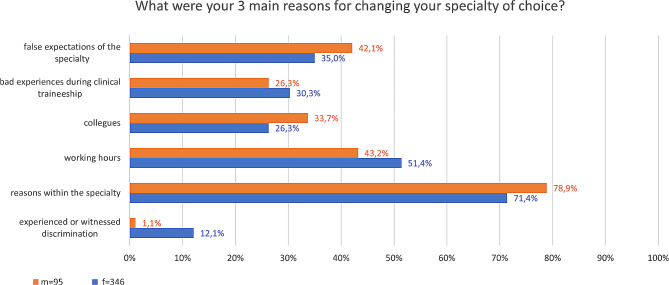
Percentages of participants selecting a reason as one of their 3 main reasons for their change of specialty

Gender discrimination was the reason for *n* = 50 (10.6%) women and *n* = 3 (2.3%) men to not consider a specialty as a career option in the first place (Table 2). The main reason for changing the initially desired speciality were working hours (f: 51.4%, m: 43.2%) and false expectations of the specialty (f: 35%, m: 42.1%) (Fig. 3). The only statistically significant difference between men and women in terms of changing the speciality was gender discrimination. Women chose gender discrimination to be among their three main reasons for a chance of a previously chosen specialty significantly more often than men (f = 42, 12.1% vs. m = 1, 1.1%, *p* = 0.0013) (Fig. 3). Altogether, *n* = 6 (1.7%) of the women reported that gender discrimination was the only reason for changing the desired speciality. None of the men in the study reported that gender discrimination was the only reason for changing their speciality choice.

**Fig. 4:**
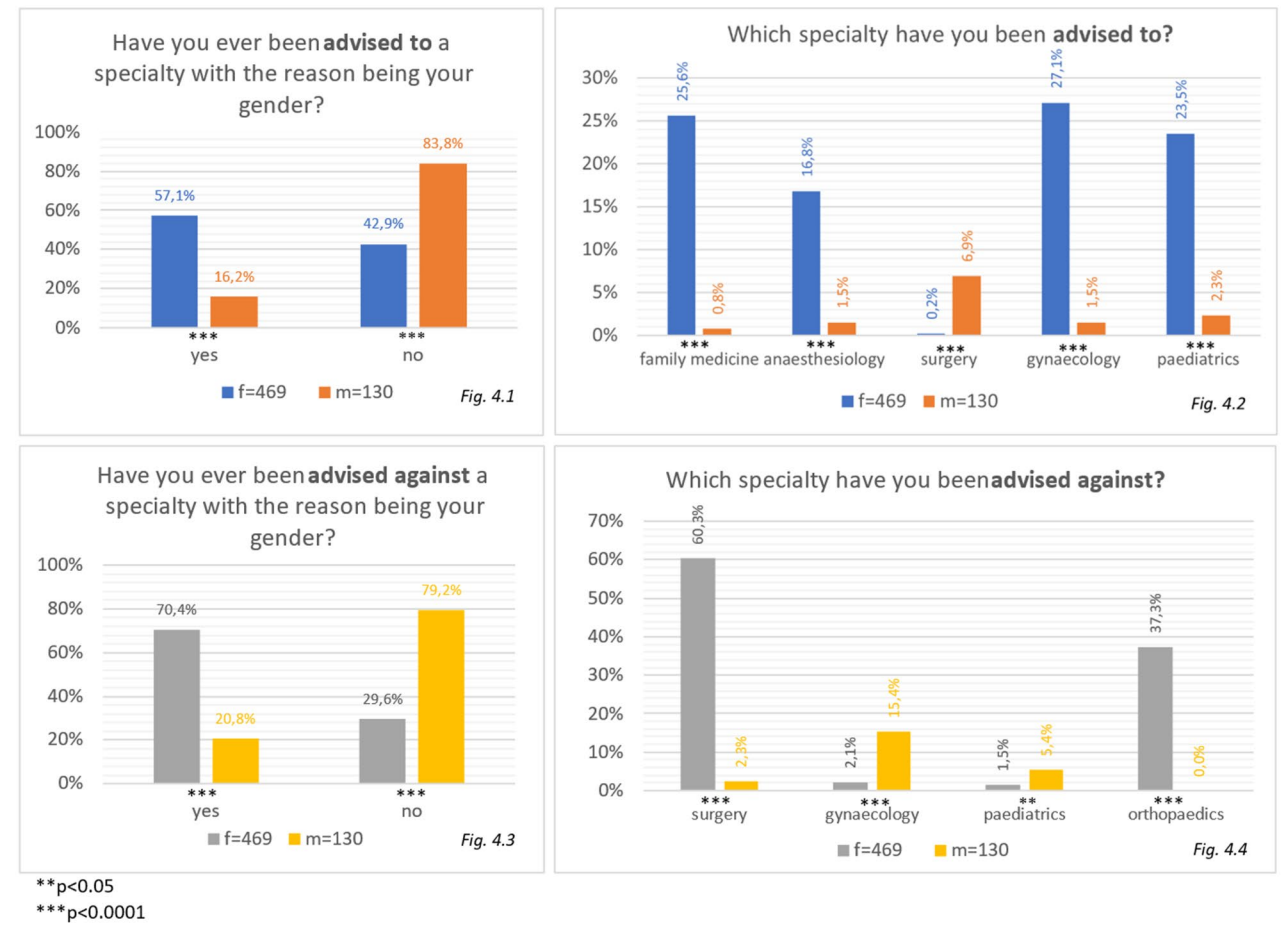
Percentages of participants that have ever been advised to or against a certain specialty, analysed by gender

*N* = 268 (57.1%) women received advise to choose a certain specialty only because of their gender compared to *n* = 21 male students (16.2%, *p* < 0.0001)(Fig. 4.1). Even more women were told not to pursue a career in a certain specialty because of their gender (*n* = 330, 70.4%, Fig. 4.3). Male students did also experience more advice against certain specialties, that toward others (against: *n* = 27, 20.8% vs. towards: *n* = 21, 16.2%). Men reported significantly more often (m = 9, 6.9% vs. f = 1, 0.2%, *p* < 0.0001) to have been advised to choose the specialty of surgery, because of their gender and not to start their career in the specialties of gynaecology (m = 20, 15.4% vs. f = 10, 2.1%, *p* < 0.0001) or paediatrics (m: *n* = 7, 5.4% vs. w: *n* = 7, 1.5%, *p* = 0.0093) (Fig. 4.2 and 4.4), while women were told to avoid surgery (f = 283, 60.3% vs. m = 3, 2.3%, *p* < 0.0001) and orthopaedics (f = 175, 37.3% vs. m = 0, 0%, *p* < 0.0001) (Fig. 4.2 + 4.4) (Fig. 4).

## Discussion

Our Germany wide survey showed that gender discrimination is a present part for medical students during their training. In comparison to a single centred study in Germany in 2020 [10], the participants at our study reported experienced or witnessed gender discrimination more frequently. The reasons for that are diverse. First, the presence of gender discrimination in all forms of media has even increased in the past 3 Years. Second, our study population was approximately two times larger and we only included 5th and 6th year students in our study population. Especially the ladder point might be of significance because students who are more advanced in their medical education might naturally have had more situations were a discrimination incident could occur.

The aggressors of gender discrimination were people that have a high standing in hospital’s hierarchy and/or the students have much contact to. That might explain that senior doctors and patients were the most frequently named aggressors. While students depend for a proper education and fair grading to a certain degree on senior doctors [14], patients as aggressors might reflect that gender discrimination is not only a problem of medical education, but of society, as well. Similar aggressor distribution was found in a Swedish study amongst final year medical students [12].

Male students showed to have significantly higher career intentions, either reaching for higher positions [10] or declaring good career options to be an important factor for the future specialty, which was confirmed in another study [16]. Interestingly a good work-life-balance was most often considered to be important for both men and women, what might seem counterintuitive. This leads to the question whether men and women define a good work-life-balance equally, or whether the goal of a good work-life-balance has become less of a gender-based goal, but more of a demand of this generation.

The gender of students that have witnessed gender discrimination more frequent were surprisingly men. Reasons could include the tendency of women to downplay occurred gender discrimination incidents, when confronted repeatedly with a hostile environment [14, 18]. This hypothesis is plausible especially in terms of the high quantities of gender discrimination during medical education [10, 12, 15, 18, 19] and that makes it quite unlikely for medical students to have never ever witnessed gender discrimination. By giving a definition of our understanding of gender discrimination and microaggressions, we tried to minimise the influence of different perceptions of discrimination on the reported incidents. However, a definition might not exclude that influence in total, meaning the subjective perception of gender discrimination remains an important bias factor that has to be considered when interpreting our results.

While gender discrimination is not one of the most frequently reported reasons, it still influenced the specialty choice of in particular for women. These findings are contrary to a similar study with medical students in the USA, that observed men to be more perceptible of experienced gender discrimination [9]. In both studies the influence of gender discrimination on the students’ specialty choice was determined by self-assessment by the participants and is therefore a subjective evaluation. Furthermore, the participants in our study were given multiple examples for reasons that might have affected their choice against one specialty, instead of just asking about the influence of the gender discrimination on their decision. The participants of a Swedish single centre study reported, that an important coping mechanisms of gender discrimination was the avoidance of unpleasant situations, people and places [14]. This might imply avoiding a certain speciality due to experiences gender discrimination.

The questionnaire also included advises the students were given concerning their future specialty, that might have an influence on their specialty choice, as well. Interestingly, female participants were more likely to be advised to a speciality where they also saw their career future, such as gynaecology, paediatrics, anaesthesiology or family medicine. Male medical students did not prefer to be surgeons as often as they were advised to. However, the advised avoidance of paediatrics and gynaecology, was similar to the preferred speciality as they were less popular among male students.

With the advice towards female students to not choose surgery or orthopaedics as a career option, the advisors named two of the specialties where most incidents of gender discrimination were reported. These findings propose a connection even though only 12.9% of students stated, that gender discrimination influenced their decision on their future speciality. Women’s predominance in obstetrics-gynaecology or paediatrics can be found across countries in various studies [6, 7, 20].

Are they still stereotypical relicts? In the case of orthopaedic surgery, women found the specialty to be unappealing because of the work/life balance and also perceived themselves as physically too weak [21]. Although there might be no objective reason for not choosing a certain physically demanding medical speciality, subjective reasons might play an important role.

In the study of Gjerberg et al. [22] the lack of flexibility in the work environment in the men dominated specialty of general surgery was observed as a further reason for not completing their training. She points out, that even though gynaecology and general surgery have similar demands on the doctors, the number of female doctors between these specialities differ a lot. This suggests that the specialties that are more attractive for women may have already found ways to be more attractive, for example by more flexible work schedules to, for example raise children.

Women have structural disadvantages when it comes to very time and capacity demanding medical specialties, because they are expected to care for their family, house and children on top of their work [4, 23]. In Germany for example, partners with unequal incomes have tax advantages.- Structurally intended the work distribution becomes unbalanced, even leading to women at work having to step back or risking burn-outs [23]. With this in mind, female medical students might choose their specialty according to their upcoming tasks in the family life.

The strong association between female medical students’ decision for a specialty and the amount of women as role models in that specialty was found by Neumayer et al. [8] indicating that the lack of role models in certain specialties, presumably for men and women, might be an important influencing factor for the students’ specialty choice, as well [21].

In contrast to the reasons for a change of the desired specialty in our analysis, other studies have identified multiple other reasons for medical students, that reportedly influenced their decision. Mohamed et all [24] found, based on a questionnaire at a Saudi-Arabian college of medicine, that the selection of specialty were economic anticipations as well as personal interest. A Slovenian study compared less subjective determinants like social backgrounds and character traits among medical students with the same specialty of choice and found resemblances among these [25]. In addition to the results in our study, a single centre study in the United Arab Emirates [20] confirmed that the atmosphere, a medical student experiences while engaging with a specialty and, to a lesser degree, the recommendations received by friends and family do affect the medical students’ decision about their specialty choice.

### Limitations

More women than men participated in our study presumable due to bias of interest in the topic, meaning the results for women are more representable than for the men. Furthermore, the amount of replies from the different universities varied a lot. Even though all of the universities were reached by at least one way of contact, the distributions’ efficiency towards students differed, so that not every German medical student matching our study population’s criteria could be reached.

With the subject of discrimination, there is always a variation on peoples’ perception of discrimination incidents and their severity. The answers to the questionnaire must be regarded as subjective descriptions. Nevertheless, it is important to acknowledge the subjective discomfort of students, as this subjective impression is, what influences decisions, such as career pathways.

## Conclusion

Gender discrimination plays an important role in medical students’ education and in choosing their striven specialty. With the increasing numbers of female doctors and the impending shortage of doctors in Germany, the unequal gender dispersion amongst the specialties, as it is now and as the participants’ current specialty choices suggest, could increase the problematic situation even further. Specialties should therefore aim to be equally attractive to both, men and women or at least to not repel genders. Other than gender discrimination it is important to establish a social structure that allows all people to combine work, family and free time in a non-overwhelming manner, so that all doctors that were thoroughly trained can work and stay healthy for a long time.

Furthermore, it should be in our societies greatest interest to train well educated doctors that choose their specialty based on their interest and talents and not because of a social role picture that was forced upon them. As in former times all medical specialities were dominated by men, it can be expected, that female doctors will also conquer currently male dominated fields.

Gender discrimination is a problem in many aspects of society and is fought by various measures. The medical faculties, as a place of education and therefore with impact on the future doctors, must support their students in addressing gender discrimination incidents. There are already Equality-Offices at most German universities, that offer assistance dealing with discrimination. Students should have low-threshold access to reporting systems for gender discrimination as well as fast help when needed.

Due to the raising awareness about gender stereotypes and discrimination, that might have been less imminent in the past, it is important as well to address the potential aggressors of such discrimination to raise their awareness on the topic, create a great understanding of it and prevent incidents from occurring.

### Electronic supplementary material

Below is the link to the electronic supplementary material.

Supplementary Material 1

## Data Availability

The study’s data is available from the corresponding author on reasonable request.
