# Supplementary material for: The effects of gender discrimination on medical students‘ choice of specialty for their (junior) residency – a survey among medical students in Germany
Source: BMC Med Educ. 2024 May 30;24:601. doi: 10.1186/s12909-024-05579-9 (PMC11140860; doi:10.1186/s12909-024-05579-9)
Supplement: Supplementary file 1 — Supplementary Material 1 [file 12909_2024_5579_MOESM1_ESM.pdf]

facharztwahlPJ → base 07.03.2023, 16:02

Seite 01

B0

Hallo!
Schön, dass du an der Umfrage teilnimmst.

Im Rahmen meiner Doktorarbeit möchte ich ermitteln, welche Faktoren für Medizinstudierende bei der Auswahl des PJ-Faches und später des Facharztes eine Rolle spielen.

Die Teilnahme an der Umfrage ist natürlich freiwillig und für alle Medizinstudierenden an deutschen Universitäten ab dem 9. Semester freigeschaltet. Ich würde mich sehr freuen, wenn du den Fragebogen im Anschluss mit deinem Freundeskreis und Kommiliton*Innen teilen würdest.

Ganz liebe Grüße aus Aachen,
Jule

Seite 02
E0

1. Studierst du an einer Universität in Deutschland Medizin?

- ◯ Ja
- ◯ Nein

2 aktive(r) Filter

Filter E001/F1
Wenn eine der folgenden Antwortoption(en) ausgewählt wurde: **2**
Dann nach dem Klick auf "Weiter" den Text **Z002** anzeigen und das Interview beenden

Filter E001/F2
Wenn eine der folgenden Antwortoption(en) ausgewählt wurde: **1**
Dann Frage/Text **E002** später im Fragebogen anzeigen (sonst ausblenden)

2. In welchem Semester deines Studiums befindest du dich?

- ◯ 1
- ◯ 2
- ◯ 3
- ◯ 4
- ◯ 5
- ◯ 6
- ◯ 7
- ◯ 8
- ◯ 9
- ◯ 10
- ◯ PJ

3 aktive(r) Filter

Filter E002/F1
Wenn eine der folgenden Antwortoption(en) ausgewählt wurde: **1, 2, 3, 4, 5, 6, 7, 8**
Dann nach dem Klick auf "Weiter" den Text **Z002** anzeigen und das Interview beenden

Filter E002/F2
Wenn eine der folgenden Antwortoption(en) ausgewählt wurde: **11**
Dann in Frage **D003** die Items **6** anzeigen (sonst ausblenden)

Filter E002/F3
Wenn eine der folgenden Antwortoption(en) ausgewählt wurde: **11**
Dann in Frage **D102** die Items **6** anzeigen (sonst ausblenden)

Seite 03
SZ

3. An welcher Universität studierst du?

[Bitte auswählen]

4. In welchem Abschnitt deines Studiums befindest du dich?

- 9. Semester
- 10. Semester
- vor 2. Staatsexamen
- nach 2. Staatsexamen
- PJ

5. Welchem Geschlecht ordnest du dich am ehesten zu?

- weiblich
- männlich
- divers

6. Wie alt bist du?

dein Alter in Jahren

7. Bist du religiös? Wenn ja, welcher Religionsgemeinschaft gehörst du an?

- Ja
- Nein

8. Was ist der höchste Bildungsabschluss deiner Eltern, bezogen auf das Elternteil mit dem höchsten Abschluss?

- Hochschulabschluss
- Fachhochschulabschluss
- Ausbildungsabschluss
- Meister*In
- Kein Abschluss
- Sonstiger
  Welcher Abschluss?

9. Welche Facharztweiterbildung / welches PJ-Wahlfach erwägst du im Moment am ehesten?

- ○ Allgemeinmedizin
- ○ Anästhesiologie
- ○ Augenheilkunde
- ○ Chirurgie
- ○ Frauenheilkunde und Geburtshilfe
- ○ Hals-, Nasen-, Ohrenheilkunde
- ○ Haut- und Geschlechtskrankheiten
- ○ Innere Medizin
- ○ Kinder- und Jugendmedizin
- ○ Neurologie
- ○ Psychosomatische Medizin und Psychotherapie
- ○ Psychiatrie und Psychotherapie
- ○ Radiologie
- ○ Orthopädie und Unfallchirurgie
- ○ Urologie
- ○ Sonstige ____________

10. Wie wichtig sind dir die Folgenden Punkte bei der Wahl für ein Fachgebiet?

geregelte Arbeitszeiten

unwichtig | weniger wichtig | wichtig | sehr wichtig

flexible Arbeitszeiten (z.B. Teilzeit)

unwichtig | weniger wichtig | wichtig | sehr wichtig

guter Verdienst

unwichtig | weniger wichtig | wichtig | sehr wichtig

gute Karrieremöglichkeiten

unwichtig | weniger wichtig | wichtig | sehr wichtig

Vereinbarkeit von Familie und Beruf

unwichtig | weniger wichtig | wichtig | sehr wichtig

neben der Krankengeschichte auch die Lebensverhältnisse der Patienten kennen

unwichtig | weniger wichtig | wichtig | sehr wichtig

ein breites Spektrum an Erkrankungen behandeln

unwichtig | weniger wichtig | wichtig | sehr wichtig

in einem Team mit Ärzten verschiedener Fachrichtungen arbeiten

unwichtig | weniger wichtig | wichtig | sehr wichtig

in einem Team mit Kollegen verschiedener Gesundheitsberufe arbeiten

unwichtig | weniger wichtig | wichtig | sehr wichtig

chirurgisch behandeln

unwichtig | weniger wichtig | wichtig | sehr wichtig

konservativ behandeln

unwichtig | weniger wichtig | wichtig | sehr wichtig

Niederlassungsmöglichkeit

unwichtig | weniger wichtig | wichtig | sehr wichtig

Geschlechtsbezogene Diskriminierung, also Diskriminierung, die auf dem Geschlecht des Opfers basiert, kann auf direkte und offensive Weise z.B. in Form von Beleidigungen oder Belästigung ausgeübt werden. Sie kann sich jedoch auch viel subtiler äußern, als sogenannte Mikroaggressionen.

„Mikroaggressionen sind alltägliche Kommentare [„ach ihr Männer/Frauen"], Fragen [„hast du dir denn Gedanken über die Familienplanung gemacht?"], verbale oder nonverbale Handlungen [Hand auflegen, tätscheln, auslachen], die überwiegend marginalisierte Gruppen treffen und negative Stereotypen verfestigen. Sie können sowohl absichtlich als auch unabsichtlich geäußert oder getätigt werden. Obwohl sie oft nicht verletzend gemeint sind, können sie dazu führen, dass sich Menschen unsicher und unwohl fühlen. Mikroaggressionen mögen im Moment klein oder unbedeutend erscheinen, aber sie summieren sich und können Menschen das Gefühl geben nicht dazuzugehören".[1]

Achtung: Im folgenden Abschnitt kommen Fragen zu Diskriminierungserfahrungen auf Grund des Geschlechts. Diese können eventuell auf Opfer von geschlechtsbezogener Diskriminierung retraumatisierend wirken.

1 (https://vielfalt.uni-koeln.de/antidiskriminierung/glossar-diskriminierung-rassismuskritik/mikroaggressionen)

11. Hast du im Rahmen deines Studiums jemals Diskriminierung aufgrund deines Geschlechts erlebt?

- ◯ sehr häufig
- ◯ häufig
- ◯ gelegentlich
- ◯ selten
- ◯ nie

1 aktive(r) Filter

Filter D002/F1
Wenn eine der folgenden Antwortoption(en) ausgewählt wurde: **5**
Dann nach dem Klick auf "Weiter" direkt zur Seite **D4** springen

12. In welchem Setting hast du diese Diskriminierungserfahrungen vor allem gemacht?
Wenn du dich nie in einem bestimmten Settings befunden hast kreuze bitte „trifft nicht zu“ an.

Universitäre Lehrveranstaltungen (Vorlesungen, Seminare, etc.)

nie	selten	gelegentlich	häufig	sehr häufig	*trifft auf mich nicht zu*

Krankenpflege Praktikum

nie	selten	gelegentlich	häufig	sehr häufig	*trifft auf mich nicht zu*

Famulaturen

nie	selten	gelegentlich	häufig	sehr häufig	*trifft auf mich nicht zu*

Praktika

nie	selten	gelegentlich	häufig	sehr häufig	*trifft auf mich nicht zu*

Arbeit auf Station (z.B. als HiWi)

nie	selten	gelegentlich	häufig	sehr häufig	*trifft auf mich nicht zu*

PJ

nie	selten	gelegentlich	häufig	sehr häufig	*trifft auf mich nicht zu*

13. Warst du jemals Zeuge von geschlechtsbezogener Diskriminierung?
(Reden über eine andere Person, Makro-/Mikroaggressionen gegenüber anderen Personen)

- ○ sehr häufig
- ○ häufig
- ○ gelegentlich
- ○ selten
- ○ nie

14. Welche Form/en von Diskriminierung hast du selbst erlebt?

Degradierende Witze oder Bemerkungen in Bezug auf dein Geschlecht

nie | selten | gelegentlich | häufig | sehr häufig

Degradierende Gesten in Bezug auf dein Geschlecht

nie | selten | gelegentlich | häufig | sehr häufig

Degradierende Spitznamen (z.B. „Schätzchen“, „Mädchen“, „Süße/r“)

nie | selten | gelegentlich | häufig | sehr häufig

Unbegründetes in Frage stellen der Fachkenntnisse

nie | selten | gelegentlich | häufig | sehr häufig

Beleidigungen oder Beschimpfungen

nie | selten | gelegentlich | häufig | sehr häufig

Bedrohungen oder Belästigung

nie | selten | gelegentlich | häufig | sehr häufig

Ungewollte Berührungen (z.B. über den Rücken streichen, Hand auflegen)

nie | selten | gelegentlich | häufig | sehr häufig

Bevorzugen von Kommiliton*Innen des anderen Geschlechts

nie | selten | gelegentlich | häufig | sehr häufig

Öffentliche Demütigung (z.B. Bloßstellen in der Visite)

nie | selten | gelegentlich | häufig | sehr häufig

Sozialer Ausschluss (z.B. Ausschluss aus Gesprächen/ gemeinsamem Mittagessen)

nie | selten | gelegentlich | häufig | sehr häufig

Ungewolltes Zeigen von pornografischem Material

nie | selten | gelegentlich | häufig | sehr häufig

Ungewollte persistierende Zuneigungen und Avancen (z.B. Flirten, private Treffen)

nie | selten | gelegentlich | häufig | sehr häufig

Unerwünschte / unpassende Komplimente

nie | selten | gelegentlich | häufig | sehr häufig

Unterschwellige sexuelle Erpressung

nie | selten | gelegentlich | häufig | sehr häufig

Sexuelle An-/Übergriffe

nie	selten	gelegentlich	häufig	sehr häufig

15. In welchen Fachgebieten hast du bereits geschlechtsbezogene Diskriminierung erfahren?

(z.B. in PJ, Praktika, Vorlesungen oder Seminaren zu diesem Fach)

Allgemeinmedizin

nie	selten	gelegentlich	häufig	sehr häufig

Anästhesiologie

nie	selten	gelegentlich	häufig	sehr häufig

Augenheilkunde

nie	selten	gelegentlich	häufig	sehr häufig

Chirurgie

nie	selten	gelegentlich	häufig	sehr häufig

Gynäkologie und Geburtshilfe

nie	selten	gelegentlich	häufig	sehr häufig

Hals-, Nasen-, Ohrenheilkunde

nie	selten	gelegentlich	häufig	sehr häufig

Dermatologie

nie	selten	gelegentlich	häufig	sehr häufig

Innere Medizin

nie	selten	gelegentlich	häufig	sehr häufig

Pädiatrie

nie	selten	gelegentlich	häufig	sehr häufig

Neurologie

nie	selten	gelegentlich	häufig	sehr häufig

Psychiatrie und Psychotherapie

nie	selten	gelegentlich	häufig	sehr häufig

Radiologie

nie	selten	gelegentlich	häufig	sehr häufig

Orthopädie und Unfallchirurgie

nie	selten	gelegentlich	häufig	sehr häufig

Urologie

nie	selten	gelegentlich	häufig	sehr häufig

Sonstige

nie	selten	gelegentlich	häufig	sehr häufig

16. Welche Funktionen hatten die Aggressoren?

Mehrfachauswahl möglich

- ☐ Kommiliton*Innen
- ☐ Dozierende
- ☐ Assistenzärzt*Innen
- ☐ Oberärzt*Innen
- ☐ Chefärzt*Innen
- ☐ Pflegekräfte
- ☐ OP-Personal
- ☐ MFAs oder MTAs (Medizinische Fachangestellte/ Medizinisch-technische Angestellte)
- ☐ Patient*Innen
- ☐ Sonstige __________

17. Wie häufig hast du geschlechtsbezogene Diskriminierung erlebt, bei der der/die Aggressor*In das gleiche Geschlecht hatte wie du?

gleiches Geschlecht wie du

- ◯ sehr häufig
- ◯ häufig
- ◯ gelegentlich
- ◯ selten
- ◯ nie

18. Wie häufig hast du geschlechtsbezogene Diskriminierung erlebt, bei der der/die Aggressor*In ein anderes Geschlecht hatte als du?

anderes Geschlecht als du

- ◯ sehr häufig
- ◯ häufig
- ◯ gelegentlich
- ◯ selten
- ◯ nie

1 aktive(r) Filter

Filter D008/F1
Wenn eine der folgenden Antwortoption(en) ausgewählt wurde: **1, 2, 3, 4, 5**
Dann nach dem Klick auf "Weiter" direkt zur Seite **W0** springen

19. Warst du jemals Zeuge von geschlechtsbezogener Diskriminierung?

(Reden über eine andere Person, Makro-/Mikroaggressionen gegenüber anderen Personen)

- ◯ sehr häufig
- ◯ häufig
- ◯ gelegentlich
- ◯ selten
- ◯ nie

1 aktive(r) Filter

Filter D101/F1
Wenn eine der folgenden Antwortoption(en) ausgewählt wurde: **5**
Dann nach dem Klick auf "Weiter" direkt zur Seite **W0** springen

20. In welchem Setting hast du diese Diskriminierung vor allem beobachtet?
wenn du dich nie in einem bestimmten Setting befunden hast, kreuze bitte „trifft nicht zu“ an

Universitäre Lehrveranstaltungen (Vorlesungen, Seminare, etc.)

nie | selten | gelegentlich | häufig | sehr häufig | *trifft auf mich nicht zu*

Krankenpflegepraktikum

nie | selten | gelegentlich | häufig | sehr häufig | *trifft auf mich nicht zu*

Famulaturen

nie | selten | gelegentlich | häufig | sehr häufig | *trifft auf mich nicht zu*

Praktika

nie | selten | gelegentlich | häufig | sehr häufig | *trifft auf mich nicht zu*

Arbeit auf Station (z.B. als HiWi)

nie | selten | gelegentlich | häufig | sehr häufig | *trifft auf mich nicht zu*

PJ

nie | selten | gelegentlich | häufig | sehr häufig | *trifft auf mich nicht zu*

21. In welcher Form äußerte sich die von dir beobachtete geschlechtsbezogene Diskriminierung?

Degradierende Witze oder Bemerkungen in Bezug auf das Geschlecht

nie | selten | gelegentlich | häufig | sehr häufig

Degradierende Gesten in Bezug auf das Geschlecht

nie | selten | gelegentlich | häufig | sehr häufig

Degradierende Spitznamen (z.B. „Schätzchen“, „Mädchen“, „Süße/r“)

nie | selten | gelegentlich | häufig | sehr häufig

Unbegründetes in Frage stellen der Fachkenntnisse

nie | selten | gelegentlich | häufig | sehr häufig

Beleidigungen oder Beschimpfungen

nie | selten | gelegentlich | häufig | sehr häufig

Bedrohungen oder Belästigung

nie | selten | gelegentlich | häufig | sehr häufig

Ungewollte Berührungen (z.B. über den Rücken streichen, Hand auflegen)

nie | selten | gelegentlich | häufig | sehr häufig

Bevorzugen von Kommiliton*Innen des anderen Geschlechts

nie | selten | gelegentlich | häufig | sehr häufig

Öffentliche Demütigung (z.B. Bloßstellen in der Visite)

nie | selten | gelegentlich | häufig | sehr häufig

Sozialer Ausschluss (z.B. Ausschluss aus Gesprächen/ gemeinsamem Mittagessen)

nie | selten | gelegentlich | häufig | sehr häufig

Ungewolltes Zeigen von pornografischem Material

nie | selten | gelegentlich | häufig | sehr häufig

Ungewollte persistierende Zuneigungen und Avancen (z.B. Flirten, private Treffen)

nie | selten | gelegentlich | häufig | sehr häufig

Unerwünschte / unpassende Komplimente

nie | selten | gelegentlich | häufig | sehr häufig

Unterschwellige sexuelle Erpressung

nie | selten | gelegentlich | häufig | sehr häufig

Sexuelle An-/Übergriffe

nie	selten	gelegentlich	häufig	sehr häufig

22. In welchen Fachgebieten hast du bereits Diskriminierung bezeugt?

(z.B. in PJ, Praktika, Vorlesungen oder Seminaren zu diesem Fach)

Allgemeinmedizin

nie | selten | gelegentlich | häufig | sehr häufig

Anästhesiologie

nie | selten | gelegentlich | häufig | sehr häufig

Augenheilkunde

nie | selten | gelegentlich | häufig | sehr häufig

Chirurgie

nie | selten | gelegentlich | häufig | sehr häufig

Gynäkologie und Geburtshilfe

nie | selten | gelegentlich | häufig | sehr häufig

Hals-, Nasen-, Ohrenheilkunde

nie | selten | gelegentlich | häufig | sehr häufig

Dermatologie

nie | selten | gelegentlich | häufig | sehr häufig

Innere Medizin

nie | selten | gelegentlich | häufig | sehr häufig

Pädiatrie

nie | selten | gelegentlich | häufig | sehr häufig

Neurologie

nie | selten | gelegentlich | häufig | sehr häufig

Psychiatrie und Psychotherapie

nie | selten | gelegentlich | häufig | sehr häufig

Radiologie

nie | selten | gelegentlich | häufig | sehr häufig

Orthopädie und Unfallchirurgie

nie | selten | gelegentlich | häufig | sehr häufig

Urologie

nie	selten	gelegentlich	häufig	sehr häufig

Sonstige

nie	selten	gelegentlich	häufig	sehr häufig

Seite 10
D6

23. Welche Funktionen hatten die Aggressor*Innen?
(Mehrfachauswahl möglich)

- ☐ Kommiliton*Innen
- ☐ Dozierende
- ☐ Assistenzärzt*Innen
- ☐ Oberärzt*Innen
- ☐ Chefärzt*Innen
- ☐ Pflegekräfte
- ☐ OP-Personal
- ☐ MFAs oder MTAs (Medizinische Fachangestellte/ Medizinisch-technische Angestellte)
- ☐ Patient*Innen
- ☐ Sonstige: ____________

24. Wie häufig hast du geschlechtsbezogene Diskriminierung bezeugt, bei der der/die Aggressor*In das gleiche Geschlecht hatte wie der/die Diskriminierte?

- ○ sehr häufig
- ○ häufig
- ○ gelegentlich
- ○ selten
- ○ nie

25. Wie häufig hast du geschlechtsbezogene Diskriminierung bezeugt, bei der der/die Aggressor*In ein anderes Geschlecht hatte als der/die Diskriminierte?

- ○ sehr häufig
- ○ häufig
- ○ gelegentlich
- ○ selten
- ○ nie

26. Wie oft hattest du im Verlauf deines Studiums das Gefühl, dass du dich deutlich mehr anstrengen musst, als Kommiliton*Innen des anderen Geschlechts?

- ○ sehr häufig
- ○ häufig
- ○ gelegentlich
- ○ selten
- ○ nie

27. Wie oft hast du im Verlauf deines Medizinstudiums (Lehre, Famulaturen, Praktika, etc.) das Gefühl gehabt, dass dein Geschlecht von Vorteil für dich war?

- ○ sehr häufig
- ○ häufig
- ○ gelegentlich
- ○ selten
- ○ nie

28. Wie oft hast du im Verlauf deines Medizinstudiums (Lehre, Famulaturen, Praktika, etc.) das Gefühl gehabt, dass dein Geschlecht von Nachteil für dich war?

- ○ sehr häufig
- ○ häufig
- ○ gelegentlich
- ○ selten
- ○ nie

Seite 12
U1

29. Hast du bei der Entscheidung über dein PJ-Wahlfach / Facharzt bereits von vornherein von einer bestimmten Fachrichtung abgesehen?

- ◯ Ja
- ◯ Nein

30. Hast du dich im Verlauf deines Studiums bezüglich deines PJ-Wahlfachs/ Facharztwunsches schon mal umentschieden?

- ◯ Ja
- ◯ Ja, mehrmals
- ◯ Nein

2 aktive(r) Filter

Filter U002/F1
Wenn eine der folgenden Antwortoption(en) ausgewählt wurde: **1, 2**
Dann Frage/Text **U003** später im Fragebogen anzeigen (sonst ausblenden)

Filter U002/F2
Wenn eine der folgenden Antwortoption(en) ausgewählt wurde: **3**
Dann nach dem Klick auf "Weiter" direkt zur Seite **R** springen

Seite 13
U0

31. Waren geschlechtsbezogene Diskriminierungserfahrungen, z.B. durch Abwertung oder sexuelle Belästigung der Grund für deinen Wechsel?

- ◯ Ja
- ◯ unter Anderem
- ◯ Nein

32. Welches sind / waren deine Hauptgründe für den Wechsel deines PJ-Wahlfachs / Facharztwunsches?

bitte gib bis zu 3 Hauptgründe an

- ☐ Erlebte / beobachtete geschlechtsbezogene Diskriminierung
- ☐ Fachliche Gründe
- ☐ Arbeitszeiten
- ☐ Kollegium
- ☐ Schlechte Erfahrungen während Famulaturen
- ☐ Schlechte Erfahrungen im PJ
- ☐ Falsche Vorstellungen vom Fach
- ☐ Persönliche / familiäre Gründe
- ☐ sonstige ____________

Seite 14

R

33. Wurde dir schon einmal auf Grund deines Geschlechts zu einem bestimmten Fach geraten? Wenn ja, zu welchem Fach und von wem?

Mehrfachauswahl möglich

- ☐ Ja
- ☐ Nein

34. Wurde dir schon einmal auf Grund deines Geschlechts geraten ein bestimmtes Fach nicht zu wählen? Wenn ja, von welchem Fach wurde dir abgeraten und von wem?

Mehrfachauswahl möglich

- ☐ Ja
- ☐ Nein

Letzte Seite

Vielen lieben Dank für deine Teilnahme an der Umfrage! Du hast mir damit sehr geholfen. Ich würde mich sehr freuen, wenn du die Umfrage mit deinem Freundeskreis und Kommiliton*Innen teilst. Ganz liebe Grüße aus Aachen, Jule

Jule Stock, Uniklinik RWTH Aachen – 2023
